# An Energy–Economic–Environment Tri-Objective Evaluation Method for Gas Membrane Separation Processes of H_2_/CO_2_

**DOI:** 10.3390/membranes14010003

**Published:** 2023-12-21

**Authors:** Junjiang Bao, Shuai Li, Xiaopeng Zhang, Ning Zhang

**Affiliations:** 1 State Key Laboratory of Fine Chemicals, Dalian University of Technology, Dalian 116023, China; baojj@dlut.edu.cn (J.B.); lishuai0123@mail.dlut.edu.cn (S.L.); xiaopengzhang@dlut.edu.cn (X.Z.); 2 School of Chemical Engineering, Dalian University of Technology, Panjin 124221, China

**Keywords:** gas membrane separation process, H_2_-selective membrane, CO_2_-selective membrane, two-stage hybrid membrane system, pre-combustion carbon capture

## Abstract

For pre-combustion carbon capture, the high syngas pressure provides a sufficient mass transfer driving force to make the gas membrane separation process an attractive option. Comparisons of combined different membrane materials (H_2_-selective and CO_2_-selective membranes) and membrane process layouts are very limited. Especially, the multi-objective optimization of such processes requires further investigation. Therefore, this paper proposes 16 two-stage combined membranes system for pre-combustion CO_2_ capture, including 4 two-stage H_2_-selective membrane systems, 4 two-stage CO_2_-selective membrane systems, and 8 two-stage hybrid membrane systems. A tri-objective optimization method of energy, economy, and environment is proposed for comprehensive evaluation of the proposed systems. Results show that with the targets of 90% CO_2_ purity and recovery, six gas membrane separation systems could be satisfied. After further multi-objective optimization and comparison, the C1H2-4 system (the hybrid system with H_2_-selective membranes and CO_2_-selective membranes) has the best performance. Feed composition and separation requirements also have an important influence on the multi-objective optimization results. The effects of selectivity and permeance of H_2_-selective and CO_2_-selective membranes on the performance of the C1H2-4 system are also significant.

## 1. Introduction

As a greenhouse gas, CO_2_ capture is of great significance to climate change. Current CO_2_ capture methods mainly include pre-combustion CO_2_ capture, oxy-combustion, and post-combustion capture [1]. Pre-combustion CO_2_ capture methods mainly include absorption [2], adsorption [3], and membrane separation methods [4]. One of the characteristics of pre-combustion CO_2_ capture is that the main products of syngas undergoing a water-gas shift (WGS) reaction are 40–60 mol% CO_2_ and 40–60 mol% H_2_ [5,6] at pressures higher than 55 bar. The high partial pressures of H_2_ and CO_2_ provide sufficient driving force across the membrane, which results in lower energy consumption of the membrane separation process and makes it an alternative method for pre-combustion CO_2_ capture. The main tasks in the design of a membrane separation process include the selection of membrane materials, the design of the process structure, and the optimization of operating parameters.

As far as membrane materials are concerned, they are mainly divided into H_2_-selective membranes and CO_2_-selective membranes [7]. For H_2_-selective membranes, Giordano et al. [8] conducted a techno-economic study of the membrane separation process with H_2_-selective membranes. Results showed that the IGCC efficiency penalty was about 5% and the CO_2_ capture cost was estimated at 16.6 €/t CO_2_ at a feed pressure of 70 bar for the membrane. Furthermore, a sensitivity analysis of operation pressure and membrane separation properties was also conducted. Gazzani et al. [9] evaluated Pb-based H_2_ selective membranes in pre-combustion CO_2_ capture systems with thermodynamic and economic considerations and showed that the cost of CO_2_ avoided was 36 €/t CO_2_.

For CO_2_-selective membranes, Han and Winston Ho [10] designed a single-stage membrane process with CO_2_-facilitated transport membranes (FTMS), mainly focused on the carrier saturation phenomenon for FTMS, and developed a homogeneous reactive diffusion model to account for it. The authors found that to reach CO_2_ purity of 95% and H_2_ recovery of 95%, a 50 CO_2_/H_2_ selectivity with an onset fugacity for carrier saturation of 10 bar for FTMS is needed. Grainger et al. [11] used a CO_2_-selective polyvinylamine membrane to reach 85% CO_2_ recovery and 95 CO_2_ purity and found that the efficiency penalty could be about 10% points. Lin et al. [12] studied the hybrid process of membrane and cryogenic process with CO_2_-selective polaris membrane. The effects of CO_2_/H_2_ selectivity and CO_2_ capture rate on the CO_2_ capture cost were researched. 

Some researchers considered the H_2_-selective membranes and CO_2_-selective membranes simultaneously. Miandoab et al. [6] developed a rigorous model accounting for various non-ideal behaviors of membrane modules. The influences of non-ideal behaviors on both H_2_-selective membranes and CO_2_-selective membranes are discussed in detail. Xu et al. [5] studied the single-stage and two-stage membrane processes with both H_2_-selective membranes and CO_2_-selective membranes for H_2_/CO_2_ separation of pre-combustion CO_2_ capture. The authors showed that a single-stage membrane process with two types of membranes could not meet the separation requirement. A two-stage membrane system with CO_2_-selective membranes performed better than that with H_2_-selective membranes in terms of H_2_ purity and recovery. From the literature above, most researchers used H_2_-selective membranes and CO_2_-selective membranes independently and few researchers have considered the hybrid system with H_2_-selective membranes and CO_2_-selective membranes.

The optimization research of system layout and operation conditions for pre-combustion CO_2_ capture is very limited. Giordano et al. [8] considered different membrane process layouts and operation conditions and optimized them to obtain the minimum CO_2_ capture cost. Our previous study [13] presented a superstructure method to simultaneously optimize the system structure and operation conditions for H_2_/CO_2_ separation. The objective function was also CO_2_ capture cost. Although the optimizations of pre-combustion CO_2_ capture systems are few, the other gas membrane separation processes (post-combustion CO_2_ capture [14,15], biogas upgrading [16,17], and so on) could bring many beneficial inspirations and suggestions. Mores et al. [18] presented a single-objective optimization work for H_2_ purification from mixtures with a two-stage membrane process. The authors used three different objective functions: total membrane area, total power demand, and total annual cost. Results showed that the different operation conditions were obtained for different objective functions. Yun et al. [19] conducted a simulation-optimization process for absorption-based and membrane-based CO_2_ capture from the iron and steel industry. A genetic algorithm optimization was used to achieve this single-objective optimization with both energy consumption and annualized cost of the CO_2_ capture as objective functions. Single-objective optimization can only deal with separation cost or energy consumption. However, a low separation cost and energy consumption are more desired for the gas membrane separation process and little research concerning this issue has been conducted. For example, Yuan et al. [20] performed a multi-objective optimization of nitrogen-selective membrane processes of N_2_/CO_2_ separation for post-combustion CO_2_ capture. Energy consumption and membrane area are considered, and optimal system layout and operation conditions could be determined after optimization. 

The combined H_2_-selective and CO_2_-selective membrane processes are less concerned but can still benefit from the advantages of H_2_-selective and CO_2_-selective membrane processes, according to the aforementioned literature study. There are very few comparisons of different membrane materials (H_2_-selective and CO_2_-selective membrane) paired with different membrane process designs; further research is required, particularly on the multi-objective optimization of such processes. As a result, 16 two-stage combined membranes system, including 4 two-stage H_2_-selective membrane systems, 4 two-stage CO_2_-selective membrane systems, and 8 two-stage hybrid membrane systems, are proposed in this work for pre-combustion CO_2_ capture. For a thorough assessment of the suggested systems, a tri-objective optimization approach of energy, economy, and environment is suggested.

## 2. Descriptions of the Process

**Figure 1:**
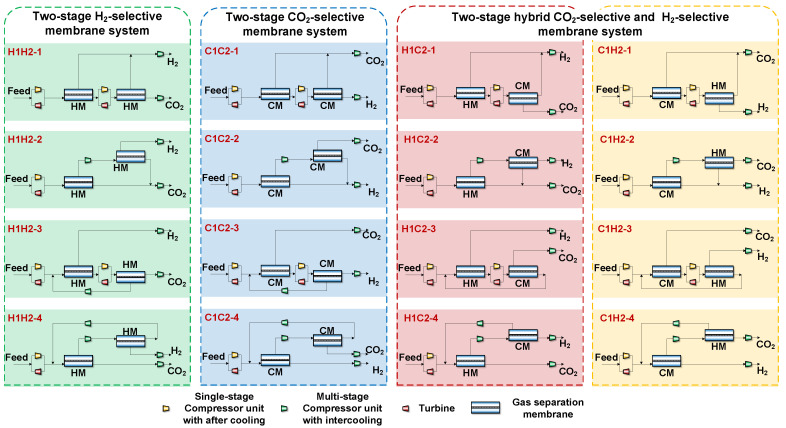
Membrane separation system with 16 different combinations of membrane materials.

In an integrated gasification combined cycle (IGCC) power plant, the separation of CO_2_ and H_2_ from shifted syngas is the main topic of this study. There are two distinct membrane types—H_2_-selective and CO_2_-selective membranes—for the H_2_/CO_2_ separation system. Since H_2_ and CO_2_ as products usually require pressurized operation, using CO_2_-selective membranes offers the advantage of producing a higher purity CO_2_ product with a lower CO_2_ pressure. While employing H_2_-selective membranes has the advantage of obtaining CO_2_ from the residual side at high pressure, the CO_2_ product’s purity is poorer as compared to using CO_2_-selective membranes. It is anticipated that using both H_2_-selective and CO_2_-selective membranes will allow for the simultaneous production of high-purity and high-pressure CO_2_ products, hence decreasing the H_2_/CO_2_ separation system’s energy requirements and membrane area. Therefore, the combination of various membrane materials and the optimization of membrane performance are required as the optimization factors are essential elements in membrane process design. As a result, 16 distinct membrane separation systems have been proposed as illustrated in Figure 1, based on the two-stage membrane separation procedure and various combinations of H_2_-selective and CO_2_-selective membranes.

As can be seen from Figure 1, in terms of the membrane separation process structure, four different membrane separation structures are considered: two-step membrane separation system without recycle (H1H2-1,C1C2-1,H1C2-1,C1H2-1), two-stage membrane separation system without recycle (H1H2-2,C1C2-2,H1C2-2,C1H2-2), two-step membrane separation system with recycle (H1H2-3,C1C2-3,H1C2-3,C1H2-3), and two-stage membrane separation system with recycle (H1H2-4,C1C2-4,H1C2-4,C1H2-4). 

At the inlet of each membrane stage, a compressor or expander configuration is used to optimize the operating pressure of the membrane if the flow is either feed or residual gas from the previous membrane stage. If the operating pressure of the membrane rises, the compressor is used to feed the membrane; otherwise, the expander is used to feed the membrane.

## 3. Materials and Methods

### 3.1. Membrane Separation Unit Modeling and Membrane Materials

An accurate description of the membrane unit has an important impact on the membrane separation process. In this paper, a discrete model [21] is used to simulate the membrane unit, which is widely used in the simulation of membrane separation processes, details of which can be found in our previous work [13] and Figure S1 of the Supporting Information. To verify the accuracy of the adopted model, it is validated in Section 4.1.

For the gas membrane separation process, the selection of the membrane material is crucial. The membrane material mainly includes inorganic [22,23], polymeric [24], and the mixed matrix membranes [25]. Compared with the other two kinds of membranes material, polymeric membranes are considered in this study because they are cheap and easily fabricated. The polymers used in this study are H_2_-selective (300 GPU H_2_ permeance, 15 H_2_/CO_2_ selectivity), and CO_2_-selective (1000 GPU CO_2_ permeance, 11.76 CO_2_/H_2_ selectivity) by MTR [26,27]. The membrane selective layer was expected to have a thickness of 1 μm, which is a typical value that can be accomplished on an industrial scale [8]. Additionally, Section 4.5 explores the impact of the membrane material’s selectivity and permeance.

### 3.2. System Performance Indexes

Numerous system performance indicators, such as production purity and recovery, total membrane area, total energy consumption, specific production cost, CO_2_ emission, and others, can be used to assess the performance of a membrane separation system. These performance indexes can be divided into three categories: energic index, economic index, and environmental index. The following are the system performance indices used in this study:

(1) CO_2_ purity

(1) $$Purity_{CO_{2}}=y_{CO_{2}\;production,\;CO_{2}}$$

where $y_{CO_{2}\;production,\;CO_{2}}$ is the mole fraction of CO_2_ in the CO_2_ production stream.

(2) CO_2_ recovery

(2) $${\mathrm{Recovery}}_{CO_{2}}=\frac{y_{CO_{2}\;production,\;CO_{2}}V_{CO_{2}\;production}}{y_{feed,\;CO_{2}}V_{feed}}$$

where $V_{CO_{2}\;production}$ is the mole flow rate of the CO_2_ production stream, $V_{feed}$ is the mole flow rate of the feed stream, and $y_{feed,CO_{2}}$ is the mole fraction of CO_2_ in the feed stream.

(3) Specific CO_2_ energy consumption SEC

This is an energic index and can be expressed by the total energy consumption divided the total captured CO_2_. The total energy consumption $W_{comsumption}$ can be calculated as follows:

(3) $$W_{comsumption}=\sum W_{compressor,i}-\sum W_{expander}$$

where $W_{compressor,i}$ is the compression work of the ith compressor, and $W_{expander}$ is the expansion work of the expander if it exists.

Specific CO_2_ energy consumption SEC can be calculated:

(4) $$SEC_{CO_{2}}=\frac{W_{comsumption}}{y_{CO_{2}\;production,\;CO_{2}}V_{CO_{2}\;production}}$$

(4) Specific CO_2_ capture cost SCC

This is an economic index and can be expressed the total cost of membrane separation system divided the total captured CO_2_. The total cost of membrane separation system mainly includes the investment cost of membrane units, compressors, turbines, and heat exchangers. The detailed calculation equations and method can be referred to our previous work [13]. Specific CO_2_ capture cost SCC can be expressed as the following equation:

(5) $$SCC_{CO_{2}}=\frac{{\mathrm{Cost}}_{total}}{y_{CO_{2}\;production,\;CO_{2}}V_{CO_{2}\;production}}$$

(5) Specific CO_2_ emission SE

This can be considered an environmental index and can be calculated by the total CO_2_ emission divided the total amount of hydrogen separated. The total CO_2_ emission mainly includes two parts: one part is the uncaptured CO_2_ in the separated H_2_ stream, and the other part is the indirect CO_2_ emission caused by the electricity consumption.

Therefore, total CO_2_ emission EMI_total_ can be calculated as

(6) $$EMI_{total}=y_{H_{2}\;production,\;CO_{2}}V_{H_{2}\;production}+W_{comsumption}E_{CO_{2}}$$

where $y_{H_{2}\;production,\;CO_{2}}$ is the mole fraction of CO_2_ in the H_2_ production stream, $V_{H_{2}\;production}$ is the mole flow rate of the H_2_ production stream, and $E_{CO_{2}}$ is the emission factor of grid electricity [28], with a value of 0.986 kg/kWh.

Specific CO_2_ emission SE can be calculated as follows:

(7) $$SE_{CO_{2}}=\frac{{EMI}_{total}}{y_{H_{2}\;production,\;H_{2}}V_{H_{2}\;production}}$$

### 3.3. Simulation and Optimization Details

The simulation of the 16 membrane separation processes mentioned in Section 2 is conducted using Aspen Hysys software V11. Aspen Hysys has accurate thermodynamic model and fast convergence rate, which can deal with the membrane separation process well. But there is no membrane operation unit in Aspen Hysys software, an in-house developed membrane unit is compiled and integrated into Aspen Hysys. This paper discusses an Integrated Gasification Combined Cycle (IGCC) power plant with a capacity of 556 MW [5,6]. The flow rate of the shifted syngas is 28,390 kmol/h, and it operates at a pressure of 3000 kPa and a temperature of 40 °C. The composition of the shifted syngas is 60% H_2_ and 40% CO_2_.

The multi-objective optimization program (gamultiobj) in Matlab is utilized for the optimization portion of this paper to achieve the optimization of objective functions. The modified Non-dominated Sorting Genetic Algorithm (NSGA-II), which is frequently employed in multi-objective optimization problems in process simulation and design, is the foundation of this application.

A two-step optimization strategy is suggested in this paper to produce the optimal membrane separation system. The CO_2_ purity and recovery (Equations (1) and (2)) are used as two objective functions in the first stage because not all membrane separation system that have been offered can produce high levels of CO_2_ purity and recovery. The detailed optimization of the energy, economy, and environment is useless and unable to satisfy the separation requirements. The membrane separation process would not be included in this stage if it cannot recover and purify CO_2_ to the required levels. The remaining membrane separation systems are then optimized using three objective functions—specific CO_2_ energy consumption, specific CO_2_ capture cost, and specific CO_2_ emission—as determined by the results of Equations (4)–(7). Following optimization, the best system can be identified by comparing all the others. The TOPSIS method of decision-making determines the optimal operating condition. TOPSIS, also referred to as Technique for Order of Preference by Similarity to Ideal Solution, is a multi-criteria decision analysis technique. It evaluates a group of options according to a predetermined standard. According to the TOPSIS, a trade-off solution is the point on the Pareto-front that is furthest from the non-ideal point and closest to the ideal point. The ideal point can be regarded as the point with the optimal performance, while the non-ideal point is the point with the worst performance, and these points are all hypothetical points. Additional information is available in previous studies [29,30].

## 4. Results and Discussion

### 4.1. Validation of the Membrane Unit

**Figure 2:**
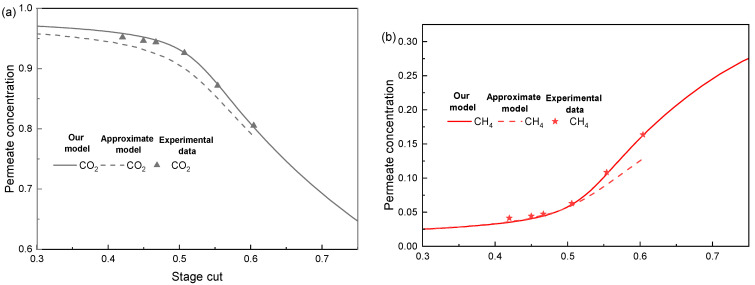

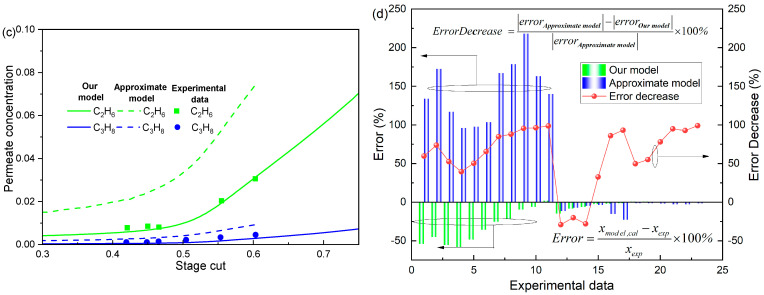
Comparison of the model in this paper with the experimental data in the literature [31] and error analysis. (**a**) Permeate concentration of CO_2_, (**b**) Permeate concentration of CH_4_ (**c**), Permeate concentration of C_2_H_6_ and C_3_H_8_, and (**d**) error analysis.

The model used in this paper was compared with experimental data from the literature [31], as shown in Figure 2, in order to confirm the accuracy of the model utilized in this paper. The experiment data for the separation of multi-component mixtures (CO_2_, CH_4_, C_2_H_6_, and C_3_H_8_) at high pressures (>35 bar) can be found in Pan’s work. Figure 2 illustrates how well the model in this research matches with experimental data from the literature at various stage cuts. Davis’ “Approximate Method” [32] was also contrasted. This method uses logarithmic mean driving force to calculate the separation performance of the membrane unit. This model, unlike ours, is unable to perform the calculation at high stage cut, and while it has a modest calculation error for high CO_2_ and CH_4_ content, it has a maximum calculation deviation of more than 220% for low C_2_H_6_ and C_3_H_8_ content (as shown in Figure 2b). Compared to the Approximate Method, the error reduction of our method is more than 50% for most of the data, and the average error reduction for all the data is 61.2%. The explanation is because, whereas our model uses a discrete model of mass transfer partial pressure for membrane separation processes, Davis’ “Approximate Method” uses the logarithmic mean partial pressure as a mass transfer driving force. Particularly for C_2_H_6_ and C_3_H_8_, our model can accomplish a more realistic representation of the mass transfer driving force.

### 4.2. Comparison of CO_2_ Purity and Recovery of Different Membrane Separation Systems

**Figure 3:**
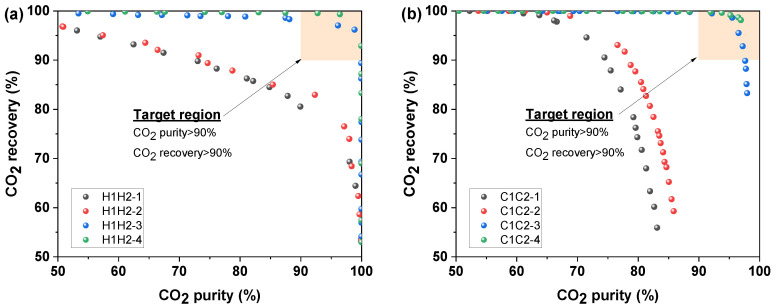

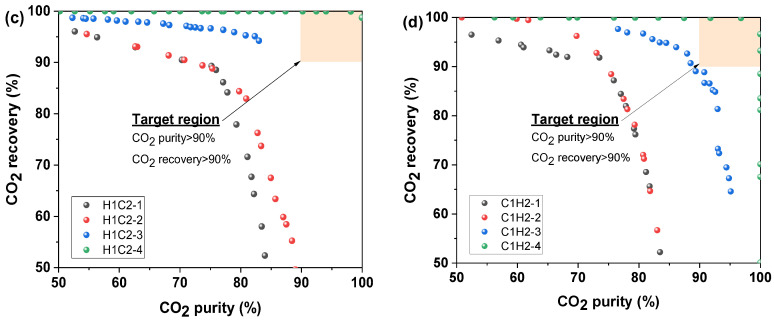
Pareto front for CO_2_ purity and recovery for different membrane separation systems. (**a**) H1H2-1,H1H2-2,H1H2-3 and H1H2-4 systems, (**b**) C1C2-1,C1C2-2,C1C2-3 and C1C2-4 systems, (**c**) H1C2-1,H1C2-2,H1C2-3 and H1C2-4 systems, and (**d**) C1H2-1,C1H2-2,C1H2-3 and C1H2-4 systems.

Since different membrane separation systems can achieve varied levels of CO_2_ purity and recovery, the 16 membrane systems are initially contrasted in terms of CO_2_ purity and CO_2_ recovery, as illustrated in Figure 3. The trade-off between CO_2_ purity and recovery is evident in Figure 3, which shows that when CO_2_ purity increases, CO_2_ recovery reduces and vice versa. High CO_2_ purity and recovery are often advantageous. For the 16 membrane systems, the membrane system with recycling has a stronger CO_2_ purity trend than the membrane system without recycling.

Following a discussion of the effects of separation requirements, this part aims to achieve 90% CO_2_ purity and 90% CO_2_ recovery, as indicated by the orange target region in Figure 3. From Figure 3, only six membrane systems—Systems H1H2-3, H1H2-4, C1C2-3, C1C2-4, H1C2-4, and C1H2-4—fall into this region, and the other membrane systems are not further examined since they could not satisfy the CO_2_ separation requirements.

### 4.3. Comparison of Six Membrane Separation Systems That Have Met Separation Requirements

Six membrane separation systems were identified to meet the demands of CO_2_ 90% purity and 90% recovery from the previous section. The CO_2_ separation energy, separation cost, and specific CO_2_ emission, as explained in Section 3.2, are employed as three objective functions to carry out the multi-objective optimization to further assess these six membrane separation systems. These six membrane separation systems were divided into three groups based on similar system architecture to facilitate comparisons: H1H2-3 vs. C1C2-3, H1H2-4 vs. C1C2-4, and H1C2-4 vs. C1H2-4. The first and second groups both make use of the same kind of membranes: H_2_-selective or CO_2_-selective membranes. The third group employs a combination of H_2_-selective and CO_2_-selective membranes.

**Figure 4:**
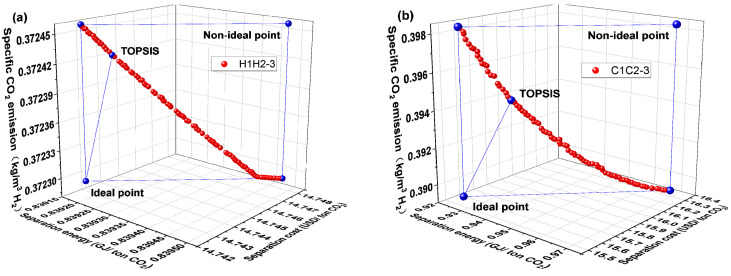
Pareto front and optimal design point for H1H2-3 and C1C2-3 membrane separation systems. (**a**) H1H2-3 system and (**b**) C1C2-3 system.

In Figure 4, the Pareto front for the H1H2-3 and C1C2-3 membrane separation systems is displayed, along with the TOPSIS decision-making method’s optimal design point. The H1H2-3 membrane separation system has lower CO_2_ separation energy, separation cost, and specific CO_2_ emission compared to the C1C2-3 membrane separation system, as can be observed from the optimal design point and Pareto front. Meanwhile, Figure 4 shows that the H1H2-3 membrane separation system, at 90% purity and 90% recovery of CO_2_, has reduced CO_2_ separation energy, separation cost, and specific CO_2_ emission. Since the permeation rate of the carbon membrane used in this paper is more than three times higher than that of the hydrogen membrane used, the range of values of these three objective functions is noticeably smaller than that of the C1C2-3 membrane separation system, implying a smaller feasible domain.

**Figure 5:**
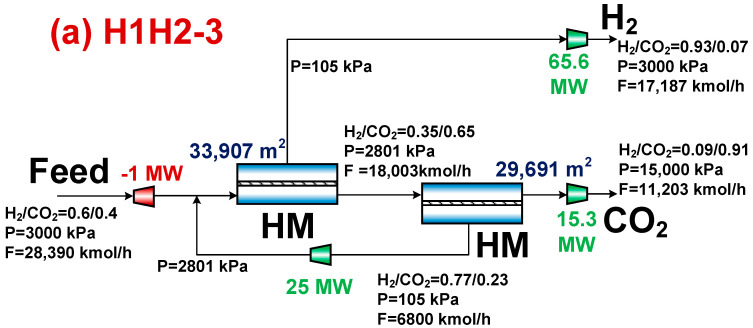

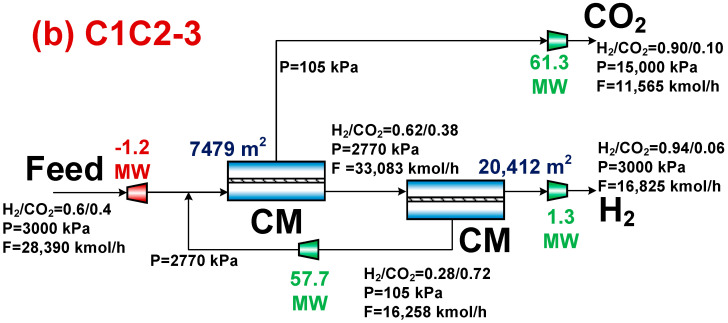
Results corresponding to the optimal design points for H1H2-3 and C1C2-3 membrane separation systems. (**a**) H1H2-3 system and (**b**) C1C2-3 system.

Figure 5 displays the membrane area, compressor power consumption, molar flow rate, and composition at the key point for the associated operational conditions at the optimal design point. According to Figure 5, even though the H1H2-3 membrane separation system’s membrane area is greater than the C1C2-3 membrane separation system’s membrane area because of the latter’s low permeation rate, the H1H2-3 membrane separation system’s compressor energy consumption is lower than that of the C1C2-3 membrane separation system, which results in a lower CO_2_ separation energy and cost. The H1H2-3 membrane separation system uses less compressor energy than the C1C2-3 membrane separation system because its recycled stream flows at a lower rate, which results in less compressor power being used. The H1H2-3 membrane separation system has a higher molar flow rate of hydrogen, which is accompanied by a lower specific CO_2_ emission and a smaller indirect CO_2_ emission caused by the compressor’s energy consumption, as shown in Equation (7).

**Figure 6:**
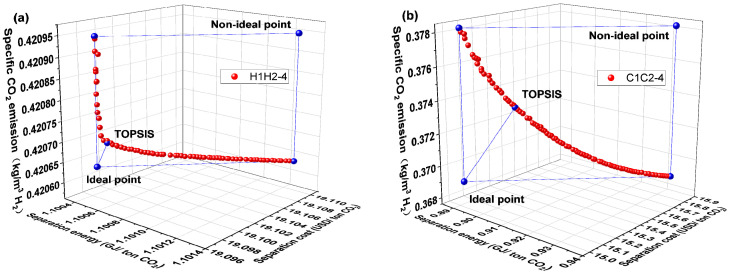
Pareto front and optimal design point for H1H2-4 and C1C2-4 membrane separation systems. (**a**) H1H2-4 system and (**b**) C1C2-4 system.

**Figure 7:**
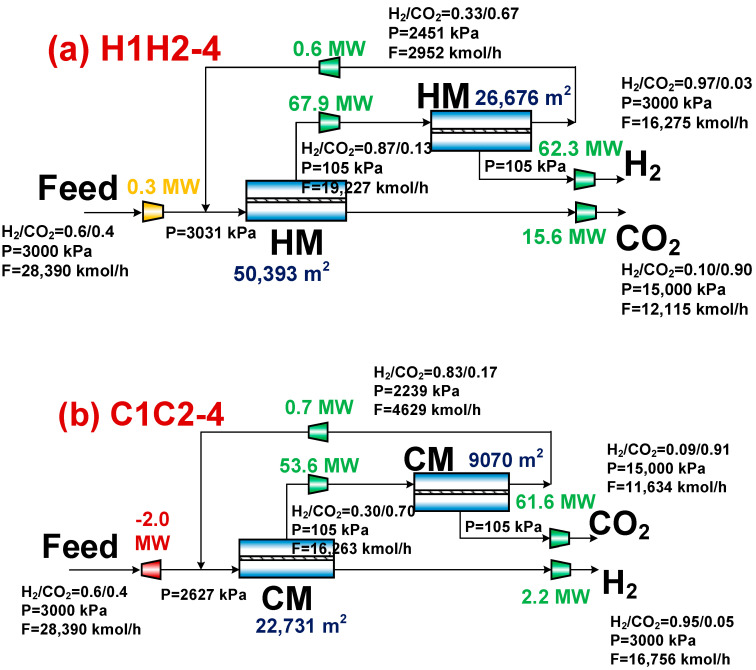
Results corresponding to optimal design points for H1H2-4 and C1C2-4 membrane separation systems. (**a**) H1H2-4 system and (**b**) C1C2-4 system.

Figure 6 displays the Pareto front and optimal design points for the H1H2-4 and C1C2-4 membrane separation systems. The C1C2-4 membrane separation system has reduced CO_2_ separation energy, separation cost, and specific CO_2_ emission compared to the H1H2-4 system, in contrast to the H1H2-3 and C1C2-3 membrane separation systems. The working conditions corresponding to the optimal design points in Figure 7 explain this. According to Figure 7, the C1C2-4 membrane separation system has a smaller total membrane area and compressor energy consumption than the H1H2-4 membrane separation system, which results in a lower CO_2_ separation energy and cost. Due to the hydrogen product of the C1C2-4 membrane separation system being derived from the residual side of the first-stage membrane, the compressor’s energy consumption is low. The small total membrane area is attributed to the C1C2-4 membrane separation system’s higher permeate rate and lower secondary membrane area, which is brought on by the secondary membrane’s smaller gas processing capacity. The specific CO_2_ emission is lower as stated in Equation (7), which is also attributable to the higher molar flow rate of hydrogen product in the H1H2-4 membrane separation system and the lower indirect CO_2_ emission caused by the compressor energy consumption.

**Figure 8:**
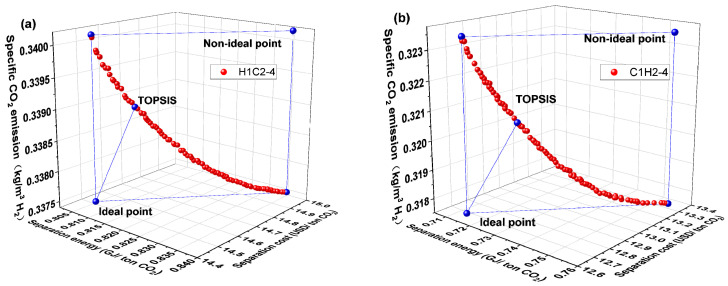
Pareto front and optimal design point for H1C2-4 and C1H2-4 membrane separation systems. (**a**) H1C2-4 system and (**b**) C1H2-4 system.

Figure 8 depicts the Pareto front and optimal design point for the H1C2-4 and C1H2-4 membrane separation systems. The C1H2-4 membrane separation system has a lower evaluation index than the H1C2-4 membrane separation system, as can be observed in Figure 8. The C1H2-4 membrane separation system, on the other hand, has the lowest CO_2_ separation energy, separation cost, and specific CO_2_ emission among all membrane separation systems, according to a thorough evaluation of Figure 4, Figure 6 and Figure 8 which is summarized in Table 1 (the optimal performance indexes by TOPSISI of six membrane separation systems). The literature [33] states that the CO_2_ separation cost of the traditional absorption technologies, such as Selexol and Rectisol, had CO_2_ separation energies of 1.121 and 1.265 GJ/ton CO_2_, respectively. The CO_2_ separation energy consumption of the C1H2-4 membrane separation system at the optimal design point is 0.725 GJ/ton CO_2_, which is 42.7% and 35.3% lower than that of the conventional absorption methods (Selexol and Rectisol), respectively, illustrating the advantages of the multi-objective optimization and the combination of membrane materials.

**Figure 9:**
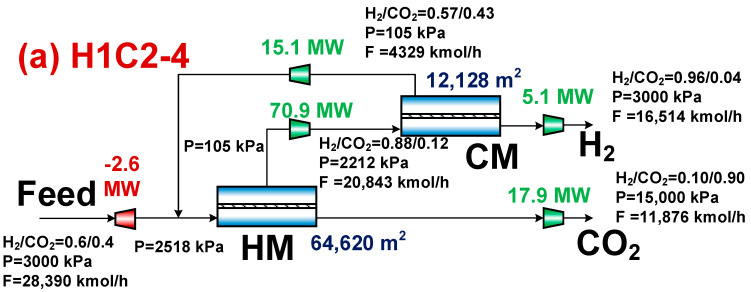

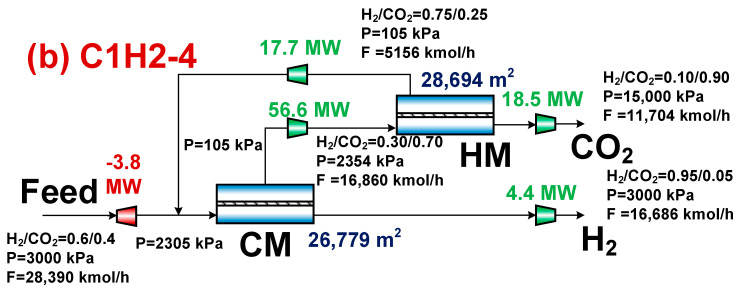
Results corresponding to optimal design points for H1C2-4 and C1H2-4 membrane separation systems. (**a**) H1C2-4 system and (**b**) C1H2-4 system.

The membrane area of the C1H2-4 membrane separation system is not the lowest (higher than that of the C1C2-3 and C1C2-4 membrane separation systems), but its compressor energy consumption is the lowest among all the membrane separation systems, which in turn results in the lowest evaluation index. This can be seen by comparing the corresponding operating conditions of the optimal design points in Figure 5, Figure 7 and Figure 9. Due to the fact that both the hydrogen product and the CO_2_ product are acquired on the residual side of both membranes, the C1H2-4 membrane separation system has the lowest compression energy consumption. Compared to the H1C2-4 membrane separation system, the use of CO_2_-selective membranes with higher permeance in the first-stage of the C1H2-4 membrane separation system results in a smaller first-stage membrane area and, in turn, a lower gas capacity for the second stage membrane, resulting in a significant reduction in compressor energy consumption between membranes.

### 4.4. Influence of Feed Composition and Separation Requirements

Six membrane separation systems that fulfill the separation requirements are contrasted in Section 4.3. The analysis demonstrates that the C1H2-4 system performs the best overall. This section examines the impact of feed composition on system performance as well as the requirement of separation.

#### 4.4.1. Effects of Feed Composition

**Figure 10:**
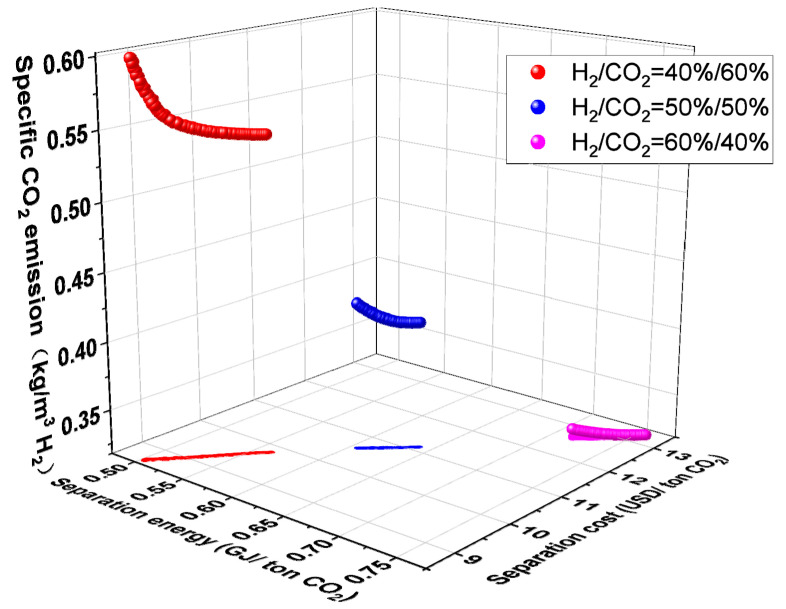
Influence of feed composition on the results of multi-objective optimization of C1H2-4 membrane separation system.

Figure 10 illustrates how feed composition affects the C1H2-4 system’s performance. As can be seen from Figure 10, as the molar fraction of H_2_ in the feed stream steadily rises, the specific CO_2_ emission falls while the CO_2_ separation energy and cost rise. This is primarily because, for a certain separation requirement, a gradual increase in the molar fraction of H_2_ in the feed implies a decrease in the molar fraction of CO_2_ in the feed and a reduced mass transfer driving force, increasing the system’s total membrane area. Greater compressor energy consumption may result from an increased flow rate brought on by a bigger membrane area. From Equations (4) and (5), increasing the total membrane area and compressor energy consumption increases the numerator in Equation (5) while decreasing the mole fraction of CO_2_ in the feed increases the denominator in Equation (4). As a result, the mole fraction of H_2_ in the feed increases the CO_2_ separation energy and separation cost. For the specific CO_2_ emission, the decrease in CO_2_ mole fraction in the feed decreases the direct indirect CO_2_ emission, although the increase in the separated H_2_ flow rate increases the indirect CO_2_ emission caused by the increase in compressor energy consumption. It is also evident from Equation (7) that the specific CO_2_ emission decreases with the increase of the molar fraction of H_2_ in the feed.

#### 4.4.2. Impact of Separation Requirements

**Figure 11:**
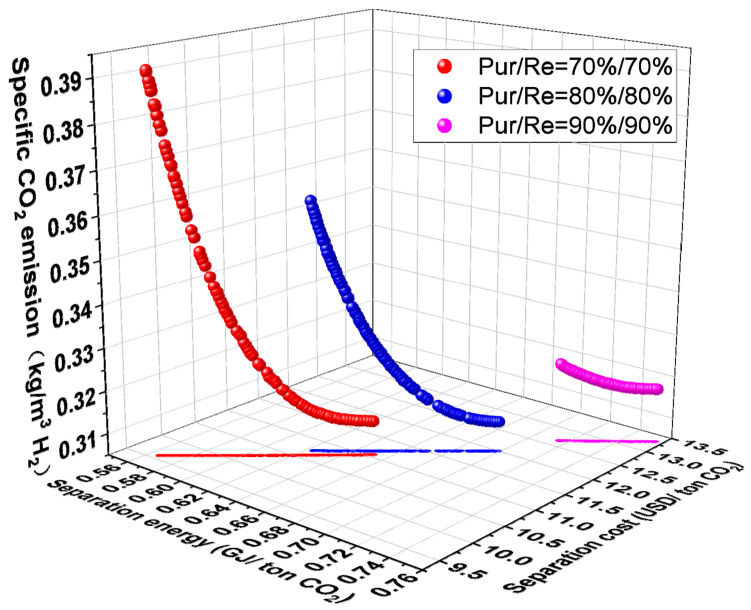
Effect of separation requirements on multi-objective optimization results of C1H2-4 membrane separation system.

Figure 11 depicts how separation requirements affect the performance of the C1H2-4 system. As shown in Figure 11, CO_2_ separation energy and cost both decrease when the separation requirements (CO_2_ purity and recovery) decrease. This is primarily because, at a fixed feed CO_2_ molar fraction, the decrease in separation requirements implies a greater mass transfer driving force, which significantly reduces the total membrane area and compressor energy consumption. Although the CO_2_ product flow is also reduced, the total cost and energy due to the total membrane area and compressor energy consumption are higher. As seen in Equations (4) and (5), as the separation requirements (CO_2_ purity and recovery) fall, so do the CO_2_ separation energy and cost. While the amount of direct CO_2_ emissions from the H_2_ product increases with the reduction of separation requirements, the amount of H_2_ in the product decreases, even though the indirect CO_2_ emission brought on by the compressor’s energy consumption declines. But overall, the amount of specific CO_2_ emission shows an elevated trend with the reduction of separation requirements.

### 4.5. Effect of Separation Performance of Membrane Materials

**Figure 12:**
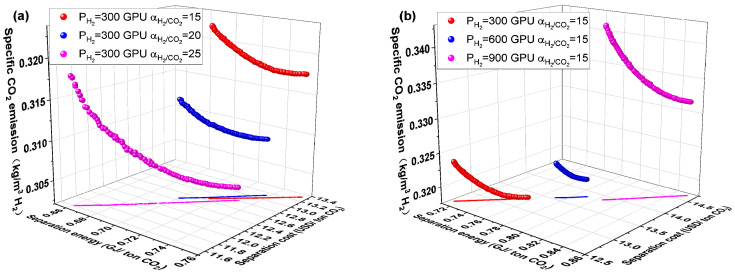
Effect of separation properties of H_2_-selective membrane on the performance of C1H2-4 membrane separation system. (**a**) H_2_/CO_2_ selectivity and (**b**) H_2_ permeance.

**Figure 13:**
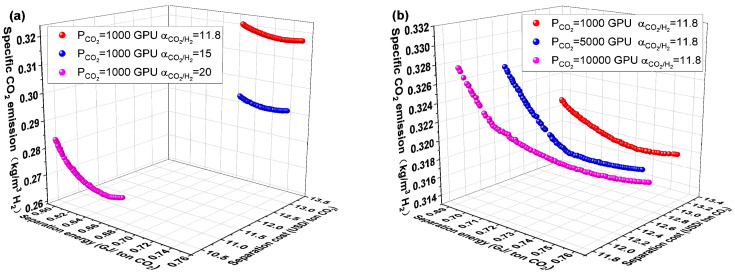
Effect of separation properties of CO_2_-selective membrane on the performance of C1H2-4 membrane separation system. (**a**) CO_2_/H2 selectivity and (**b**) CO_2_ permeance.

Membrane materials’ separation properties significantly impact membrane separation processes’ performance indices. The impacts of selectivity and permeance of H_2_-selective and CO_2_-selective membranes on the performance of the C1H2-4 system are examined in this part to examine the influence of the separation performance of membrane materials. As can be shown in Figure 12a, as the selectivity α_H2/CO2_ of the H_2_-selective membrane increased from 15 to 20 and 25, the CO_2_ separation energy, CO_2_ separation cost, and specific CO_2_ emission dropped. When the permeance of H_2_ is fixed at 300 GPU, the required membrane area is slightly increased for a fixed CO_2_ separation requirement. However, the required compressor energy consumption is reduced by a larger magnitude, which results in a reduction in CO_2_ separation energy and separation cost when the selectivity of the H_2_-selective membrane is increased. The decrease in specific CO_2_ emission is mainly due to the indirect CO_2_ emission caused by the compressor energy consumption. The performance of the C1H2-4 system is affected in the same way by the rise in selectivity α_CO2/H2_ of the CO_2_-selective membrane, as shown in Figure 13a, and for the same reasons.

The CO_2_ separation energy, separation cost, and specific CO_2_ emission all rise as the H_2_ permeance of the H_2_-selective membrane rises, as illustrated in Figure 12b. At a given **α_H2/CO2_**, the H_2_-selective membrane’s H_2_ permeance increases along with the CO_2_ permeance. The membrane area reduces as the permeance rises, but the recycled flow rate also rises. This increases the energy used by the compressor, which also raises the total amount of energy used by the compressor. Increased CO_2_ separation energy and specific CO_2_ emission result from increased overall compressor energy consumption. The cost of CO_2_ separation rises even though the cost decrease brought on by a smaller membrane area is not as much as the cost increase brought on by a higher compressor energy level.

However, the law of CO_2_ permeance of carbon membrane in Figure 13b is different from that in Figure 12b. The CO_2_ separation energy, separation cost, and specific CO_2_ emission all drop as the CO_2_ permeance of the CO_2_-selective membrane rises. The required membrane area reduces as the CO_2_-selective membrane’s CO_2_ permeance rises. Although the permeance increases along with the compressor energy consumption on the residual side of the CO_2_-selective membrane, the recycled flow rate decreases the energy consumption of the corresponding compressor, which reduces the overall compressor energy consumption. All performance indicators are decreased because of the decreased compressor power consumption and overall membrane area.

## 5. Conclusions

Sixteen two-stage mixed membrane systems, comprising four two-stage H_2_-selective membrane systems, four two-stage CO_2_-selective membrane systems, and eight two-stage hybrid membrane systems are proposed in this work for pre-combustion CO_2_ capture. A tri-objective optimization method for energy, economy, and environment is also suggested for a thorough assessment of the suggested systems. The following conclusions can be made based on the simulation and optimization of these sixteen two-stage membrane systems:

(1) The sixteen membrane systems are initially contrasted in terms of CO_2_ purity and CO_2_ recovery. The trade-off between CO_2_ purity and recovery is evident, which shows that when CO_2_ purity increases, CO_2_ recovery reduces and vice versa. To achieve 90% CO_2_ purity and 90% CO_2_ recovery, only six membrane systems—Systems H1H2-3, H1H2-4, C1C2-3, C1C2-4, H1C2-4, and C1H2-4—satisfy the CO_2_ separation requirements.

(2) The six membrane separation systems that have met separation requirements have been divide into three groups: H1H2-3 vs. C1C2-3, H1H2-4 vs. C1C2-4, and H1C2-4 vs. C1H2-4. Through the comprehensive comparison, the C1H2-4 membrane separation system has the lowest CO_2_ separation energy, separation cost, and specific CO_2_ emission among all membrane separation systems. The CO_2_ separation energy consumption of the C1H2-4 membrane separation system at the optimal design point is 0.725 GJ/ton CO_2_, which is 42.7% and 35.3% lower than that of the conventional absorption methods (Selexol and Rectisol), respectively.

(3) The impact of feed composition and the requirement of separation on the C1H2-4 membrane separation performance is studied. As the molar fraction of H_2_ in the feed stream steadily rises, the specific CO_2_ emission falls while the CO_2_ separation energy and cost rise. CO_2_ separation energy and cost both decrease when the separation requirements (CO_2_ purity and recovery) increase. However, specific CO_2_ emission shows an elevated trend with the reduction of separation requirements.

(4) The impacts of selectivity and permeance of H_2_-selective and CO_2_-selective membranes on the performance of the C1H2-4 system are examined. As the selectivity of the H_2_-selective and CO_2_-selective membranes increased, the CO_2_ separation energy, CO_2_ separation cost, and specific CO_2_ emission dropped. The CO_2_ separation energy, separation cost, and specific CO_2_ emission all rise as the H_2_ permeance of the H_2_-selective membrane rises, but the CO_2_ separation energy, separation cost, and specific CO_2_ emission all drop as the CO_2_ permeance of the CO_2_-selective membrane rises.

## Figures and Tables

**Table 1:** Optimal performance indexes by TOPSISI of six membrane separation systems.

Systems	Performance Indexes		
	Separation Energy (GJ/ton CO_2_)	Separation Cost (USD/ton CO_2_)	Specific CO_2_ Emission (kg/m^3^ H_2_)
H1H2-3	0.8392	14.7434	0.3724
C1C2-3	0.9360	15.7142	0.3945
H1H2-4	1.1005	19.0983	0.4207
C1C2-4	0.9010	15.2246	0.3735
H1C2-4	0.8148	14.5520	0.3391
C1H2-4	0.7252	12.8182	0.3207

## Data Availability

The data presented in this study are available on request from the corresponding author. The data are not publicly available due to ongoing researches using a part of the data.

## Supplementary Materials

The following supporting information can be downloaded at: https://www.mdpi.com/article/10.3390/membranes14010003/s1, Figure S1: Schematic diagram of membrane unit model (counter-current).
