# Supplementary material for: An Energy–Economic–Environment Tri-Objective Evaluation Method for Gas Membrane Separation Processes of H2/CO2
Source: Membranes (Basel). 2023 Dec 21;14(1):3. doi: 10.3390/membranes14010003 (PMC10820163; doi:10.3390/membranes14010003)
Supplement: Supplementary file 1 [file membranes-14-00003-s001.zip › membranes-2728667-supplementary.pdf]

*Supplementary material for*

# An energy-economic-environment tri-objective evaluation method for gas membrane separation processes of $H_2/CO_2$

**Junjiang Bao [1,2], Shuai Li [1,2], Xiaopeng Zhang [1,2] and Ning Zhang [1,2,*]**

The membrane unit model is established in this paper using the mathematical approach suggested by Coker et al. [1]. It is assumed that the membrane unit functions on the basis of a counter-current flow pattern represented by hollow fibers, as seen in Fig. S1. The membrane module is segmented into 100 identical area steps, and the subsequent formula determines the permeability of component *i* within a given stage:

$$m_{i,k} = Q_i \Delta A_k (P_{F_{p,k}} x_{i,k} - P_{F_{p,k}} y_{i,k})$$

where $Q_i$ is the permeance of the membrane to gas component i (kmol/(m²s • Pa)), $F_{f,k}$ and $F_{p,k}$ represent the flow rates of the feed side and permeate side of the membrane at the kth stage, and x and y are the compositions of component i in the feed side and permeate side, respectively. $m_{i,k}$ is the molar flow rate (kmol/s) of component j through the kth stage. The pressure (Pa) on the feed side and permeate side are denoted by the variables $P_{Ff,k}$ and $P_{Fp,k}$, respectively.

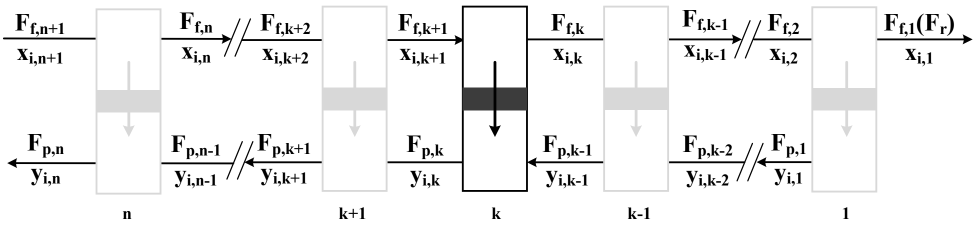


**Figure S1.** Schematic diagram of membrane unit model (counter-current)[2].

Based on the joint solution of the material conservation equation and the membrane transfer equation for each stage, it enables the solution of the molar flux and molar fraction for each stage.

The following presumptions are included in the developed model: (1) There is no loss of pressure between the residual and feed sides.(2) An isothermal temperature is used to operate the membrane.(3) There is a disregard for the concentration polarization phenomena. (4) Selectivity and permeance remain unchanged. (5) The intrinsic features of membrane transport are described by the solution-diffusion model.

Before the membrane unit model can be utilized, the mathematical model of the membrane operating unit must be assembled, converted into a DLL (dynamic link library) file, and registered in the Aspen Hysys software.

[1] D. Coker, B. Freeman, G. Fleming, Modeling multicomponent gas separation using hollow‐fiber membrane contactors, AIChE journal, 44 (1998) 1289-1302.

[2] Z. Ni *et al.*, "Synchronous Design of Membrane Material and Process for Pre-Combustion $CO_2$ Capture: A Superstructure Method Integrating Membrane Type Selection," *Membranes,* vol. 13, no. 3, p. 318, 2023.
